# Water-soluble ions and source apportionment of PM_2.5_ depending on synoptic weather patterns in an urban environment in spring dust season

**DOI:** 10.1038/s41598-022-26615-y

**Published:** 2022-12-19

**Authors:** Bowen Cheng, Yuxia Ma, Heping Li, Fengliu Feng, Yifan Zhang, Pengpeng Qin

**Affiliations:** Ministry of Education, College of Atmospheric Sciences, Key Laboratory of Semi-Arid Climate Change, Lanzhou University, Lanzhou, 730000 China

**Keywords:** Atmospheric chemistry, Environmental monitoring

## Abstract

Emission sources and meteorological conditions are key factors affecting the intensity and duration of air pollution events. In the current study, using the daily concentrations of PM_2.5_ (particulate matter with a diameter ≤ 2.5 μm) and the water-soluble ions thereof in Lanzhou from March 1, 2021, to May 31, 2021, we investigated the contributions of emission sources and locations of potential sources through positive matrix factorization and potential source contribution function analysis. In addition, synoptic weather patterns affecting pollution were typed using T-model principal component analysis. The results revealed that the average concentrations of PM_2.5_ for the entire spring, dust storm days, and normal days were 54.3, 158.1 and 33.0 μg/m^3^, respectively. During dust storm days, sulfate produced from primary emissions was mainly present in the form of K_2_SO_4_, Na_2_SO_4_, MgSO_4_, and CaSO_4_, and nitrate was mainly produced through secondary conversion and took the form of NH_4_NO_3_. Dust, industrial entities, biomass combustion, metal smelting, secondary aerosol, and sea salt contributed to 32.0, 29.8, 13.4, 11.2, 10.8 and 2.7% of the spring PM_2.5_, respectively, in Lanzhou. The main potential sources of PM_2.5_ during the normal days were in the western parts of Lanzhou. Dust storms entered Lanzhou through the Hexi Corridor from several dust sources: southeastern Kazakhstan, Mongolia, the Kurbantungut Desert, and the Badain Jaran Desert. The northwest high-pressure; northern strong high-pressure and southwest low-pressure; northwest high-pressure and southwest high-pressure synoptic weather circulation types were prone to dust storms. Our results may provide a basis for local environmental governance.

## Introduction

Atmospheric particulate matter (PM) is a mixture of direct emissions from natural and anthropogenic sources and secondary particles formed through gas-to-particle conversion processes. PM can adversely affect the local environment and human health^1–4^. Water-soluble ions are the main components of PM. Therefore, analyzing the water-soluble ions in PM can provide evidence to elucidate their source contributions, physical and chemical properties, and transportation processes^5^.

Many domestic and international studies^6–8^ analyzed the sources and components of particulate matter based on Positive Matrix Factorization (PMF) and potential source contribution function (PSCF). A PMF-based source analysis study in Athens, Greece^9^ showed that road traffic and sulfate-rich regional aerosols were the main sources of roadside traffic and urban background, respectively. A study in Lecce, southern Italy^10^ demonstrated that organic and inorganic secondary aerosols accounted for 43% of PM_2.5_ with little seasonal variation, while secondary organic carbon (SOC) was larger during cold periods and sulfate was larger in the summer. A study in Chengdu, China^11^ indicated that the main sources of PM_2.5_ are secondary aerosols and coal combustion, with the southern, southeastern and eastern Sichuan basin being the most likely potential sources of PM_2.5_.

The physical processes of air pollutants, such as their diffusion, transport, and settlement, can be influenced by weather processes, such as wind speed and direction, turbulence, and precipitation^12,13^. Weather conditions and their progression can affect the spatial distribution and temporal variation of pollutants, which can directly influence the duration and severity of the pollution process^14^. Shen et al. indicated that the Polar Jet and the Bermuda High streams considerably influence the ozone concentrations in the eastern United States^15^. Hou et al. demonstrated that uniform high-pressure and eastern high-pressure weather types were more likely to cause fine particulate pollution in China^16^. To achieve a scientific understanding of the air pollution formation mechanism and accurate air pollution forecasting, the influence of different weather conditions on pollutant transport and diffusion must be analyzed.

In the spring of 2021, China experienced its most intense and wide-reaching dust storm in 10 years, which affected most parts of northern China^17^. The storm began on the night of March 14 and continued until March 18, when it gradually disappeared, affecting the North, Northwest, Northeast, and Jianghuai regions of China^18^. Lanzhou, a key comprehensive transportation hub and industrial base in northwest China, belongs to an arid and semi-arid climatic region and is severely influenced by dust storms^6^. Lanzhou is industrialized, with petroleum and chemical refining, machinery, and metallurgy as its main industries; it has become the main production base for heavy chemicals, energy, and raw materials in China^19,20^. Because of its unique valley–basin topography, industrial structure, and geographical location, Lanzhou has become one of the most polluted cities in the country^21^. In recent years, Lanzhou has implemented measures, such as emissions reduction, dust suppression, and vehicle control programs, to control air pollution levels, which has greatly improved the air quality of the city^22^. However, the spring sandstorms remain a pollution control problem in Lanzhou.

In this study, we analyzed the mass concentration and content of water-soluble PM_2.5_ (particulate matter with a diameter ≤ 2.5 μm) ions in Lanzhou in the spring of 2021. Furthermore, we investigated the origin and sources of these ions and their contributions. Moreover, we categorized the influences of different weather types on pollution. Our study can enable an in-depth understanding of the spring atmospheric environmental quality in Lanzhou and provide a scientific basis for environmental governance and efforts to improve the environmental quality.

## Data and methods

### Sampling site and description

**Figure 1:**
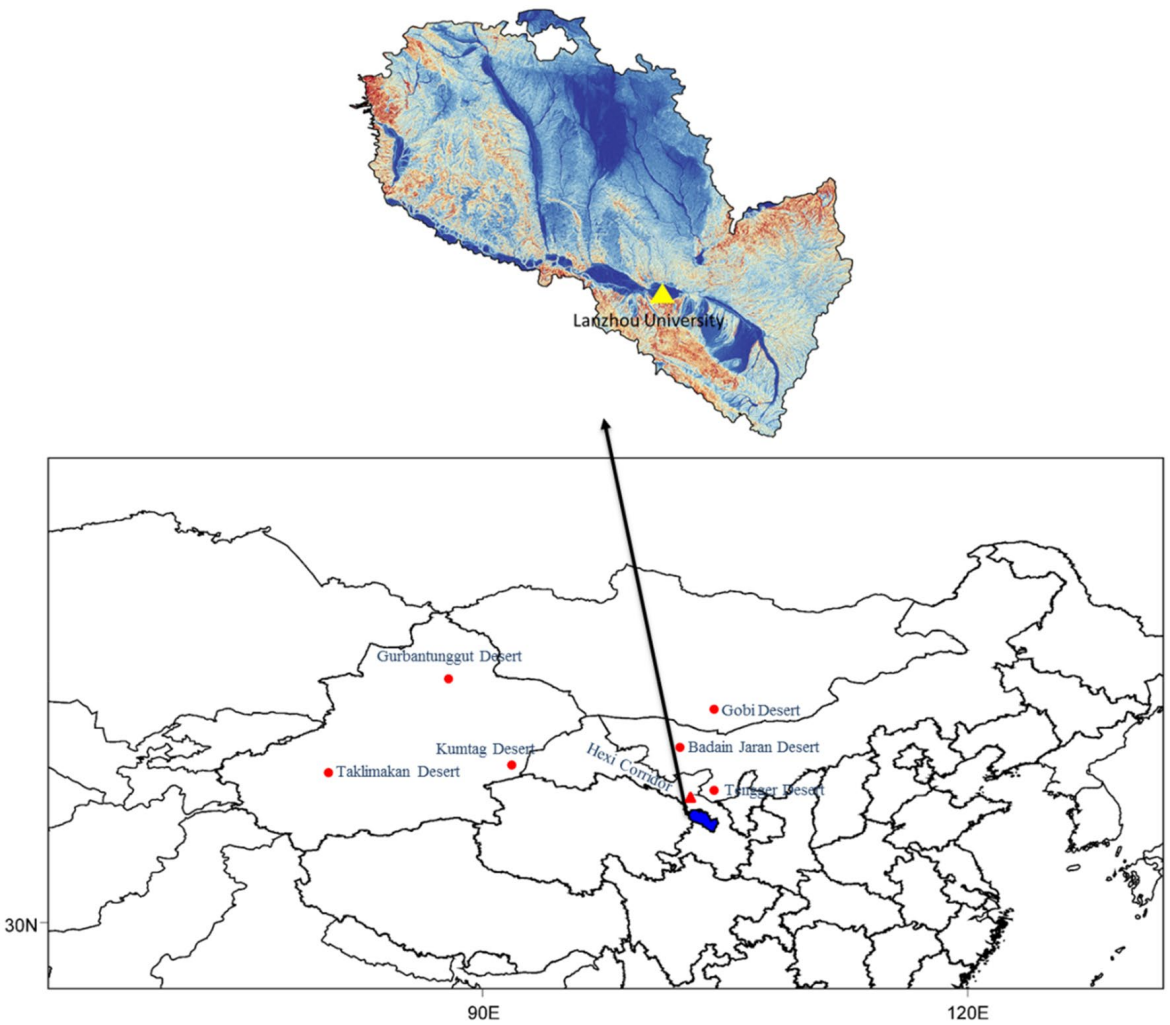
Location of sampling site (The statellite imagery was created on ArcGIS 10.3 platform (http://www.esri.com/software/arcgis/arcgis-for-desktop) and the map of Asia was created by MeteoInfo 3.1.7 (http://www.meteothink.org/downloads/index.html)).

The sampling site was an upper floor of a building (55-m height) at Lanzhou University, located in the southwest area of Lanzhou City (Fig. 1). The site is surrounded by residential areas and some shopping malls, with no significant pollution sources; the site is representative of a typical environment of mixed urban traffic, residential areas, and commercial areas. Twenty-three hourly PM_2.5_ samples (10 am to 9 am) were collected from March 1, 2021, to May 31, 2021, on a quartz filter (Whatman, 90-mm diameter) using a TH-150 medium flow sampler (Tianhong, Wuhan, China) operating at a flow rate of 100 L/min. A total of 88 samples were collected, including 15 samples on dust storm days and 73 samples on normal days.

The meteorological Physical Sciences Laboratory reanalysis data were obtained from the National Centers for Environmental Prediction (NCEP)/National Center for Atmospheric Research (NCAR) data set (https://www.psl.noaa.gov/data/gridded/data.ncep.reanalysis.html), including 850 hPa 4 × daily geopotential height reanalysis data and 850 hPa 4 × daily u-wind and v-wind reanalysis data (2.5° × 2.5° grids).

### Water-soluble ion analysis

Ultrasonic techniques were used to extract inorganic ions from the samples; typically, more than 98% of sulfate, ammonium, and nitrate ions can be extracted through ultrasonic treatment^23^. A quarter of each filter sample was immersed in a sealed vial containing 50 mL of ultrapure water and was extracted three times with ultrasonic treatment for 15 min. The water-soluble ion content in the PM_2.5_ was determined using Metrohm 925 Eco IC (Switzerland) ion chromatography. Anion (F^−^, Cl^−^, NO_3_^−^, and SO_4_^2−^) concentrations were determined using a Metrosep C 6-150/4.0 (Metrohm) column with a mixture of 3.2 mMol/L Na_2_CO_3_ and 1 mMol/L NaHCO_3_ as an eluent. Cation (Na^+^, NH4^+^, K^+^, Ca^2+^, and Mg^2+^) concentrations were determined using a Metrosep A Supp 5-150/4.0 (Metrohm) column with 4 mMol/L HNO_3_ as an eluent. The detection limits for these ions were in the range of 0.01–0.03 μg/m^3^. The samples of 7 days were selected for repeat testing and the standard error was found to be less than 5%. To evaluate the recovery efficiency of the method, the sample solution was spiked with a known amount of ions and the measurement found that the recovery was 95.6–102.0%.

### Positive matrix factorization analysis

A positive matrix factorization (PMF) receptor model was used as a multivariate factor analysis approach to analyze the contribution of different sources to the samples on the basis of the components and speciation of these sources^24^. PMF was used to sort the specific sample data matrix into factor contribution (G) and factor profile (F) matrices^25,26^. The PMF equation is as follows:

$${x}_{ij}=\sum_{n=1}^{p}{g}_{in}{f}_{nj}+{e}_{ij} ,$$

where *i* is the number of the samples, *j* represents the types of chemical species, *n* is the number of factors, *x* is the measured concentration, *g* indicates the amount of mass contributed by the factors, *f* represents the species profile of the sources, and *e*_*ij*_ represents the residual of the species.

In PMF, nonnegativity constraints are used on the matrices to reduce the rotational degrees of freedom^27^; furthermore, *Q* is minimized in the analysis process to obtain the optimal solution. The equation for *Q* is as follows:

$$\mathrm{Q}=\sum_{i=1}^{m}\sum_{j=1}^{k}{\left(({x}_{ij}-\sum_{n=1}^{p}{g}_{in}{f}_{nj})/{u}_{ij}\right)}^{2}$$

$$u\_ij=5/6*MDL x\_ij<MDL$$

$${u}_{ij}=\sqrt{{\left(RSD*{x}_{ij}\right)}^{2}+{\left(0.5*MDL\right)}^{2}*{x}_{ij} } {x}_{ij}>MDL,$$

where *u*_*ij*_ represents the estimated uncertainty values, *MDL* is the method detection limit, and *RSD* is the error fraction. In this study, the *RSD* was set to 0.1^28^. The water-soluble ion samples were added to the Environmental Protection Agency PMF 5.0 (United States Environmental Protection Agency) for analysis of the contribution of sources. The signal-to-noise (S/N) criterion was used to classify the input variables, and the species Na^+^, NH_4_^+^, K^+^, Ca^2+^, Mg^2+^, Cl^−^, NO_3_^−^, and SO_4_^2−^ were classified as “strong variables” and F^−^ as “weak variables” for which a triple uncertainty was used. And the displacement (DISP) method was used to assess the uncertainty in profiles and contribution of the estimated factors. Results showed that the drop of Q value was less than 0.1% and no factor swapped for the smallest dQmax, indicating that the results were credible.

### Potential source contribution function analysis

Potential source contribution function (PSCF) analysis is a method for identifying potential source areas of air pollution by using backward trajectory analysis^14^. In this study, reanalysis data with a resolution of 1 × 1° provided by the NCEP Global Data Assimilation System was used as the initial meteorological field. The Hybrid Single-Particle Lagrangian Integrated Trajectory model was used to calculate the backward trajectory of Lanzhou in spring. The PSCF model was based on the ratio of the cumulative probability of pollution trajectories passing through each grid to all trajectories^29^. The equation is as follows:

$$\mathrm{PSCF}=\frac{(\frac{{m}_{ij}}{N})}{(\frac{{n}_{ij}}{N})}=\frac{{m}_{ij}}{{n}_{ij}} ,$$

where *N* represents the total number of trajectory points counted in the modeling, *m*_*ij*_ represents the pollution trajectory points in the *ij* grid, and *n*_*ij*_ represents all trajectory points in the *ij* grid. In this study, the trajectories with PM_2.5_ concentrations over 35 μg/m^3^ were considered pollution tracks. The 96-h backward trajectories were calculated four times per day at 00:00, 06:00, 12:00 and 18:00 LT. The grid layer was from 30 to 60° N and 70 to 120° E, including more than 95% of the area covered by all the trajectories. And the grid size of the geographic region was designed to be 0.25 × 0.25°.

### Classification of synoptic weather patterns

Spring synoptic weather patterns were classified using the T-model principal component analysis (T-PCA) method improved upon by Huth et al.^30^ in the PCA aspect. The basic purpose of T-PCA is to sort original high-dimensional data *Z* into two low-dimensional matrices *F* and *A* (*Z* = *FA*^*T*^, with *F* as the principal component and *A* as the load). All principal components were sorted according to their contributions to the original data, and the corresponding principal component *F* was filtered and dimension reduced on the basis of the cumulative contribution of the original data^30–32^. T-PCA has temporal and spatial stability and can accurately reflect the characteristics of the original weather circulation^33^. In this study, regions 80°E to 120°E and 30°N to 60°N were selected for the analysis of prevailing weather circulation. T-PCA was performed using COST733 class software, which was developed through an initiative within the Earth System Science and Environmental Management domain of the European Cooperation in Science and Technology framework (http://cost733.met.no/).

## Results and discussion

**Figure 2:**
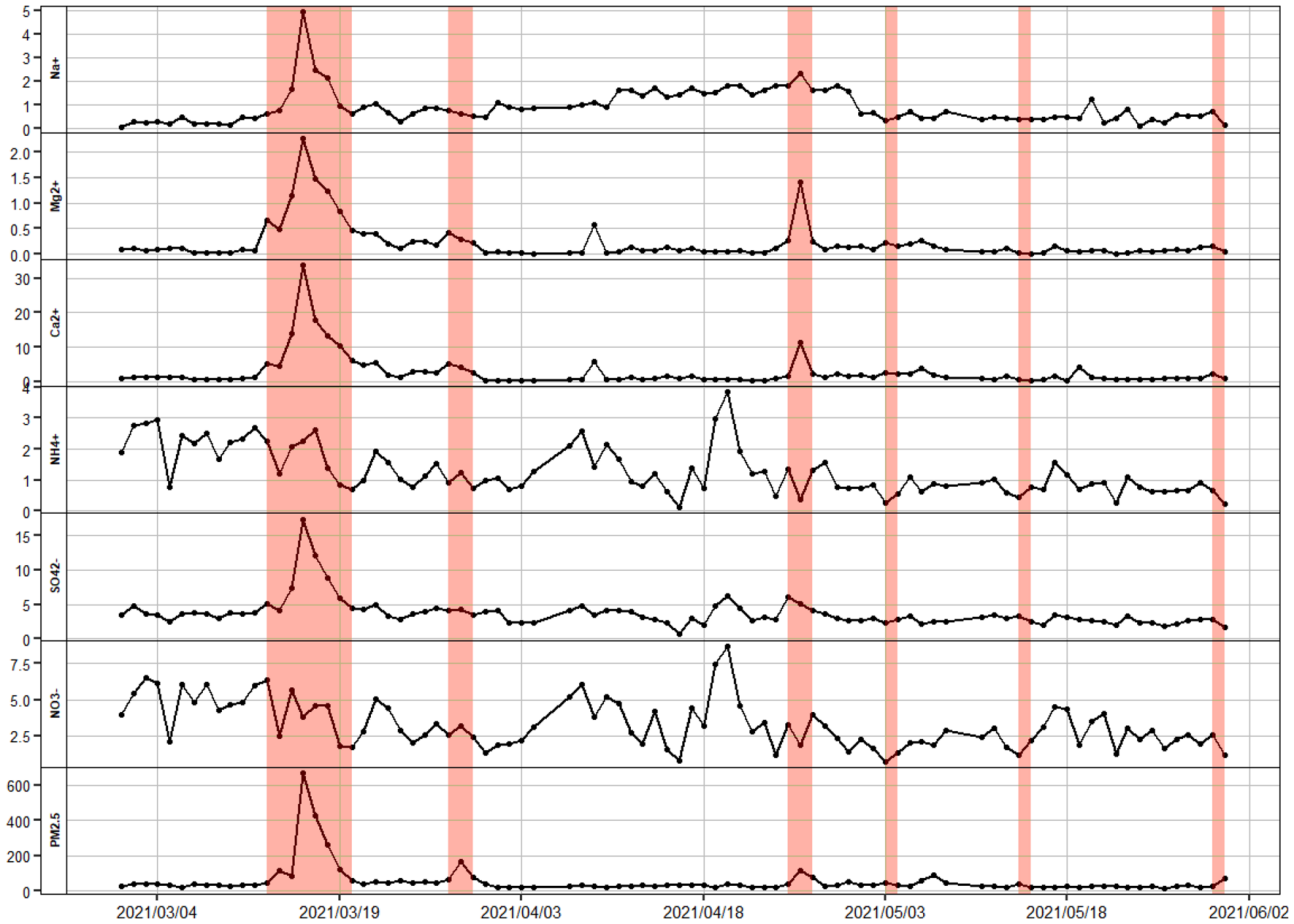
Time series of PM_2.5_ and the water-ion concentrations from March 1, 2021, to May 31, 2021, in Lanzhou, China (colored bars indicate dust storm days).

The average PM_2.5_ and water-soluble ion concentrations for whole spring and the dust storm and normal days of March 1 to May 31, 2021, in Lanzhou are listed in Table 1. The daily average PM_2.5_, Na^+^, NH_4_^+^, K^+^, Mg^2+^, Ca^2+^, F^−^, Cl^−^, NO_3_^−^, and SO_4_^2−^ concentrations for the entire spring were 54.3, 0.91, 1.28, 0.35, 0.21, 2.56, 0.10, 0.66, 3.29 and 3.87 μg/m^3^, respectively. During the dust storm days, the PM_2.5_, Na^+^, K^+^, Mg^2+^, Ca^2+^, Cl^−^, and SO_4_^2−^ concentrations increased significantly (Fig. 2), with concentrations of 158.1, 1.36, 0.59, 0.75, 8.73, 0.91 and 6.18 μg/m^3^, respectively. By contrast, the NH_4_^+^ and NO_3_^−^ concentrations decreased, with concentrations of 1.20 and 3.05 μg/m^3^, respectively. During the normal days, the average PM_2.5_ concentration was 33.0 μg/m^3^, which is lower than the China National Ambient Air Quality Standard (35 μg/m^3^). Concentrations of NH_4_^+^ and NO_3_^−^ increased, and other water-soluble ions remained at low levels. During the dust storm days, the concentration of F^−^ only increased by 0.01 μg/m^3^.

**Table 1:** Average PM_2.5_ and water-soluble ion concentrations for whole spring, dust storm days, and normal days in the spring of 2021 in Lanzhou, China.

Ions	Mean ± SD	Percentage ± SD	Dust ± SD	Normal ± SD
PM_2.5_ (μg/m^3^)	54.3 ± 85.1		158.1 ± 176.2	33.0 ± 13.2
Na^+^ (μg/m^3^)	0.91 ± 0.72	1.68 ± 1.32%	1.36 ± 1.23	0.82 ± 0.53
NH_4_^+^ (μg/m^3^)	1.28 ± 0.76	2.36 ± 1.40%	1.20 ± 0.79	1.29 ± 0.76
K^+^ (μg/m^3^)	0.35 ± 0.26	0.64 ± 0.48%	0.59 ± 0.54	0.31 ± 0.12
Mg^2+^ (μg/m^3^)	0.21 ± 0.35	0.39 ± 0.64%	0.75 ± 0.64	0.10 ± 0.10
Ca^2+^ (μg/m^3^)	2.56 ± 4.60	4.71 ± 8.47%	8.73 ± 8.76	1.30 ± 1.16
F^−^ (μg/m^3^)	0.10 ± 0.08	0.18 ± 0.15%	0.11 ± 0.07	0.10 ± 0.08
Cl^−^ (μg/m^3^)	0.66 ± 0.50	1.21 ± 0.92%	0.91 ± 0.64	0.61 ± 0.45
NO_3_^−^ (μg/m^3^)	3.29 ± 1.65	6.06 ± 3.04%	3.05 ± 1.73	3.34 ± 1.65
SO_4_^2−^ (μg/m^3^)	3.87 ± 2.12	7.13 ± 3.90%	6.18 ± 4.07	3.39 ± 0.94

SD means standard deviation.

Compared with previous years^22,34^, the concentrations of PM_2.5_ and water-soluble ions in spring 2021 had been further reduced. PM_2.5_ concentrations in 2021 spring decreased by 38.9% and 33.2% compared to 2014 and 2018^35,36^, respectively. In recent years, Lanzhou has committed to environmental management and implemented measures such as emission reduction, dust reduction and vehicle control to improve air quality^22^. And the reduction in anthropogenic emissions from industry and transportation due to COVID-19 lockdown also led to the reduction of air pollutants in Lanzhou in the spring of 2021^37^. During dust storm days, PM_2.5_ concentrations increased rapidly, as well as the concentrations of Na^+^, Mg^2+^, Ca^2+^, K^+^, Cl^−^, and SO_4_^2−^. Previous studies also showed that PMs and crustal material increased rapidly during dust storm days. A study in Xi’an^38^, northwest China, showed that Ca^2+^, SO_4_^2−^ and NO_3_^−^ were the most abundant ions in TSP samples during dust storm days, but the concentration of secondary aerosols was lower than those during normal days. A study in Riyadh, Saudi Arabia^39^ presented that PMs and some elements (such as Fe, Mg, Ca and Al) increased more than doubled during dust storm days. Shen et al. indicated that during dust storms, crustal matter and carbon-containing matter accounted for 69% and 14% of PM_2.5_, respectively^40^. However, the concentration of NO_3_^−^ and NH_4_^+^ decreased during the dust storm days. A study in Lanzhou indicated that due to the comprehensive effects of dust adsorption, the drag effect of sedimentation, and the strengthening of atmospheric diffusion capacity caused by increased wind speeds, concentrations of local emissions have decreased significantly, resulting in lower NO_3_^−^ and NH_4_^+^ concentrations^34^. A study in Seoul, South Korea^41^ demonstrated that during heavy dust periods, NO_3_^−^ concentrations decreased, while Ca^2+^ and Mg^2+^ concentrations increased significantly.

The Pearson correlations between water-soluble ions on normal and dust storm days in Lanzhou are listed in Table 2. During normal days, NH_4_^+^ was positively correlated with Cl^−^, NO_3_^−^, and SO_4_^2−^, indicating that ammonium salts were mainly present in the form of NH_4_Cl, NH_4_NO_3_, and (NH_4_)_2_SO_4_. Previous studies^42,43^ also indicated that the compositions of PM_2.5_ in different regions were consistent with our results during normal days. Gluščić et al. demonstrated that SO_4_^2−^, NO_3_^−^, and NH_4_^+^ were the main contributing ions to the total mass of PM_2.5_, and these ions were present in the atmosphere in the form of NH_4_NO_3_, NH_4_HSO_4_, and (NH_4_)_2_SO_4_ in Croatia^42^. A study performed in Heze, China^44^ showed that ions mainly exist in the form of NH_4_NO_3_, NH_4_HSO_4_, and (NH_4_)_2_SO_4_ in winter and autumn, and the forms were more complex and diverse in spring and summer. Chen et al. found that in Sichuan, China, the content of NH_4_^+^ in most samples during spring was sufficient to neutralize SO_4_^2^ and NO_3_^−^ to form (NH_4_)_2_SO_4_ and NH_4_NO_3_^45^. A study in southeastern Italy^46^ indicated there was a strong correlation between SO_4_^2-^ and NH_4_^+^, with the ammonium salt in the form of ammonium sulfate.

**Table 2:** Pearson correlations between water-soluble ions for normal and dust storm days from March 1, 2021, to May 31, 2021 in Lanzhou, China.

Days	Ions	Na^+^	NH_4_^+^	K^+^	Mg^2+^	Ca^2+^	F^−^	Cl^−^	NO_3_^−^	SO_4_^2−^
Normal	Na^+^	1.00								
	NH_4_^+^	− 0.01	1.00							
	K^+^	0.14	0.72**	1.00						
	Mg^2+^	0.09	− 0.1	0.21	1.00					
	Ca^2+^	− 0.01	− 0.11	0.24*	0.90*	1.00				
	F^−^	− 0.16	− 0.11	0.18	0.23	0.35*	1.00			
	Cl^−^	− 0.28*	0.34**	0.62**	0.24*	0.30*	0.51**	1.00		
	NO_3_^−^	0.03	0.94**	0.65**	− 0.10	− 0.11	− 0.08	0.20	1.00	
	SO_4_^2−^	0.23	0.72**	0.56**	0.22	0.15	− 0.10	0.18	0.63**	1.00
Dust	Na^+^	1.00								
	NH_4_^+^	0.55*	1.00							
	K^+^	0.91**	0.74**	1.00						
	Mg^2+^	0.92**	0.58*	0.91**	1.00					
	Ca^2+^	0.93**	0.59*	0.95**	0.96**	1.00				
	F^−^	-0.06	0.25	0.11	− 0.06	0.03	1.00			
	Cl^−^	0.70**	0.83**	0.89**	0.78**	0.79**	0.32	1.00		
	NO_3_^−^	0.38	0.89**	0.57*	0.44	0.39	0.12	0.64*	1.00	
	SO_4_^2−^	0.92**	0.72**	0.97**	0.91**	0.97**	0.08	0.84*	0.49	1.00

*p < 0.05; **p < 0.01.

During dust storm days, the correlations between SO_4_^2−^ and K^+^, Na^+^, Mg^2+^, and Ca^2+^ ions were stronger than those of NH_4_^+^, indicating that sulfate was mainly present in the form of K_2_SO_4_, Na_2_SO_4_, MgSO_4_, and CaSO_4_. A strong positive correlation was identified between NO_3_^−^ and NH_4_^+^, indicating that nitrate was mainly present in the form of NH_4_NO_3_. Wu et al. indicated that due to the numerous mirabilite ores distributed along dust transmission pathways in desert source areas of northwest China, sulfate mainly originates from primary discharge in the chemical form of Na_2_SO_4_ and CaSO_4_ during dust storm periods^47^. Wu et al. indicated that the hydrolysis of N_2_O_5_ on neutral mineral dust salts (e.g., CaSO_4_ and Na_2_SO_4_) can promote the formation of NH_4_NO_3_ during dust storm periods, leading to the accumulation of NH_4_NO_3_ on dust surfaces^48^. In this study, during normal days, a negative correlation was evident between Na^+^ and Cl^−^, whereas during the dust storm period, a significant positive correlation was observed between the two ions, indicating that the sandstorm brought sea salt aerosol (or salt water lake aerosol) through long-range transmission to Lanzhou.

**Figure 3:**
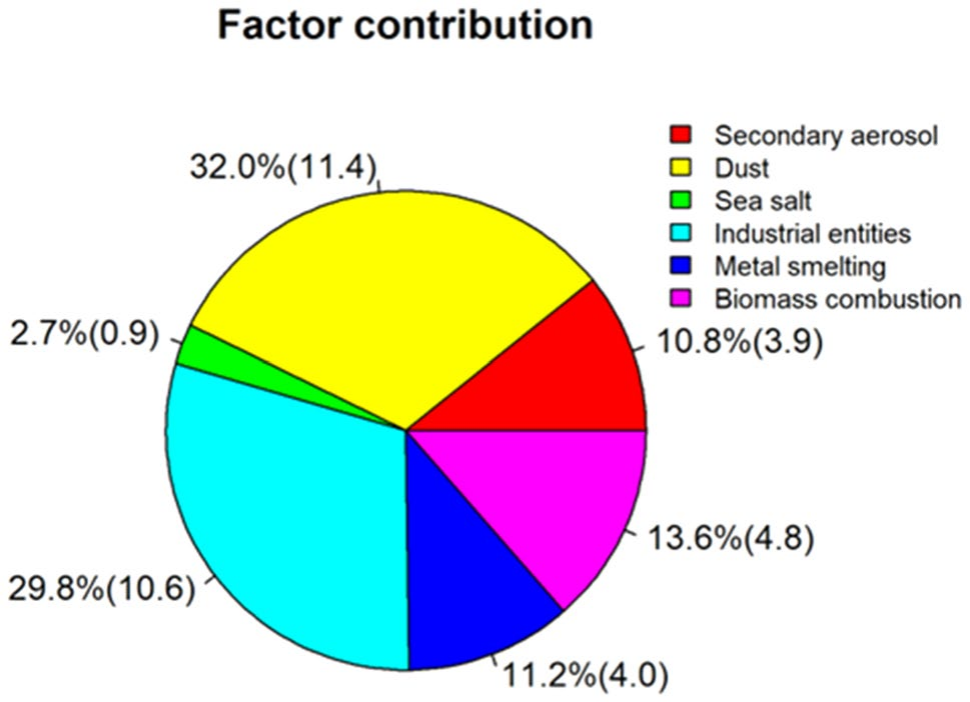
Factor contribution and mass concentration (μg/m^3^) to spring 2021 PM_2.5_ concentrations in Lanzhou, China.

**Figure 4:**
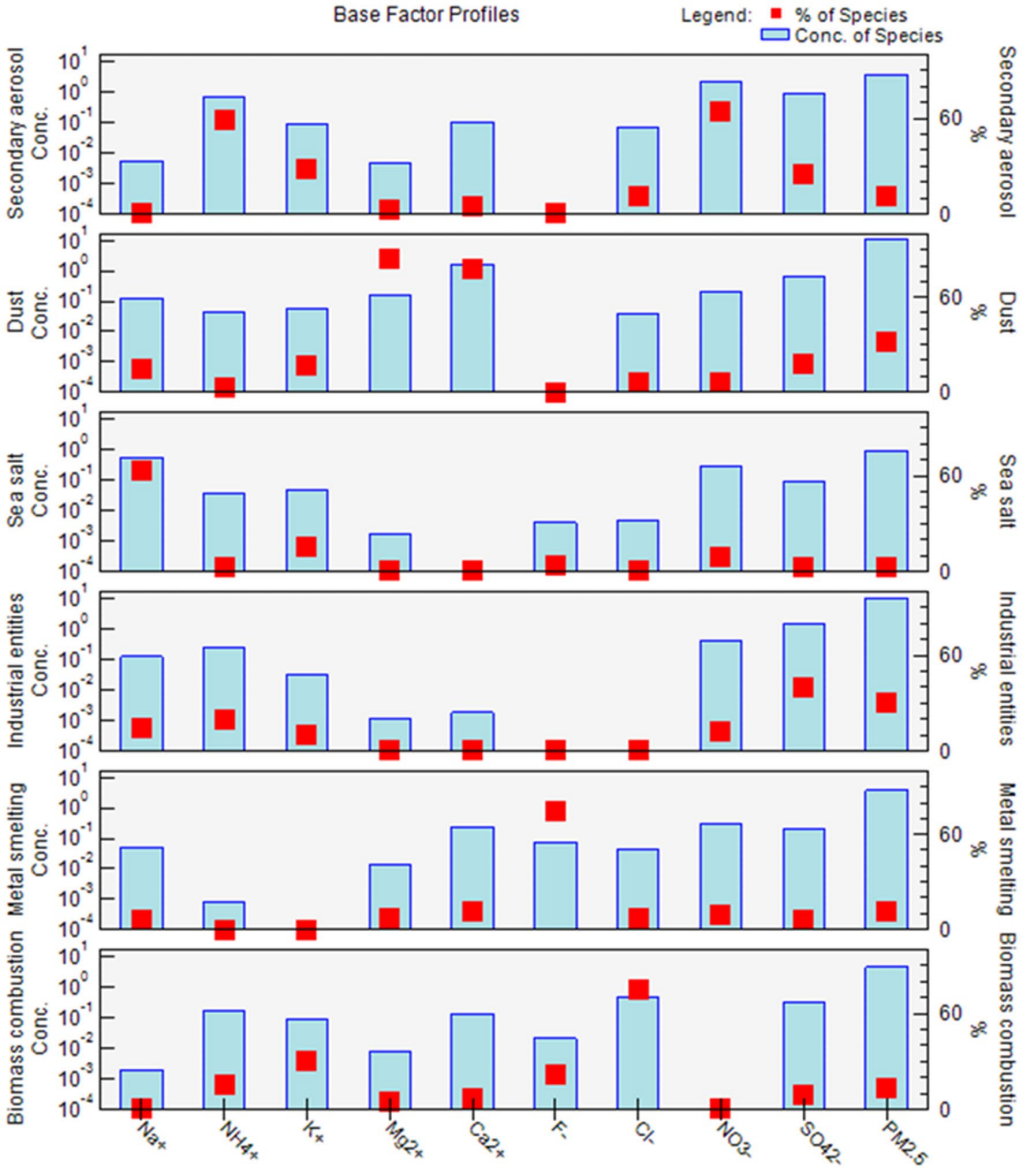
PMF analysis of spring PM_2.5_ emission source distribution in Lanzhou, China.

The main characteristic species and corresponding contributions of the six emission sources identified using the PMF model are presented in Figs. 3 and 4. Dust was the highest contributor to spring PM_2.5_ concentrations in Lanzhou, accounting for 32.0%. The main characteristic water-soluble ions with a dust source were Ca^2+^, Mg ^2+^, and SO_4_^2−^. Lanzhou is located on the northeastern edge of the Tibetan Plateau, with the Taklamakan, Kumtag, and Gurbantunggut deserts to the west and the Badain Jaran and Tengger deserts to the north^49^, leading to frequent dust storms in spring. The second highest emission source contributing to PM_2.5_ concentrations was industrial entities, accounting for 29.8%. The main characteristic water-soluble ions with industrial entities source were SO_4_^2−^, NO_3_^−^, NH_4_^+^, and Na^+^. The volatilized chemical solvents and produced pollutants from large industrial sources (such as power plants, petrochemical plants, oil refineries, and pharmaceutical plants), located in the upper winds of Lanzhou, were transported to the observation area, resulting in a high concentration of industrial emissions^50^. The contribution of biomass combustion to PM_2.5_ concentrations was 13.4%, and the main water-soluble ions with this source were K^+^ and Cl^−^. The contribution of metal smelting to PM_2.5_ concentrations was 11.2%, and the main water-soluble ions with this source were F^−^, SO_4_^2−^, NO_3_^−^ and Ca^2+^. Lanzhou is a major aluminum production base in China, and the electrolysis of aluminum produces F^−^, resulting in large amounts of F^−^ from metal smelting sources^51^. The contribution of secondary aerosol to PM_2.5_ concentrations was 10.8%, which was mainly characterized by high concentrations of NH_4_^+^, SO_4_^2−^ and NO_3_^−^. Lanzhou is located in the Yellow River valley basin, surrounded by mountains^52^. This unique basin topography prevents air pollutants from being easily diffused, and the long-term retention of these pollutants facilitates the formation of secondary aerosols^52,53^. Sea salt (or salt water lake aerosol) contributed the least to PM_2.5_ concentrations, only accounting for 2.7%. The main characteristic water-soluble ions with a sea salt source were Na^+^ and K^+^. In addition, correlation analysis revealed that sea salt aerosol (or salt water lake aerosol) was brought to Lanzhou through the long-range transport of dust storms. Na^+^, Cl^−^, K^+^ and Mg^2+^ are the main ions of sea salt aerosol^54^. Reaction of gaseous or aqueous HNO_3_ or H_2_SO_4_ with NaCl in sea salt or oxidation of gaseous SO_2_ to sulfuric acid by sea salt droplets leads to chloride depletion in sea salt aerosols^25^. A study in Shenzhen, China also reported that the aged sea salt was indicated by high loadings of Na^+^ and Mg^2+^ and low concentration of Cl^−56^. A study in Lijiang, China indicated that sea salt aerosol contains 37% of K^+^^56^.

Due to the difference of industrial structure and climate in different regions, the sources contribution of pollutant is also different. PMF results obtained from Gwangju, Korea^57^ indicated that the contributions of secondary nitrate, secondary sulfate and traffic emissions are the main sources of PM_2.5_, accounting for 26, 23 and 14%, respectively. A study in Rabigh, Saudi Arabia^58^ showed that soil, fossil-fuel combustion, industrial dust, vehicular emissions and sea sprays contributed 39.9, 19.9, 14.7, 13.4 and 12.1% to ambient PM_2.5_, respectively. Studies performed in southern cities in China, such as Nanjing, Chengdu and Chongqing^45,59^, indicated that secondary aerosols contributed the most to PM_2.5_, which might be related to local meteorological factors such as humidity and wind speed. A study in Taipei, China^60^ demonstrated that industrial entities contributed 40.0% to PM_2.5_, mainly characterized by high concentrations of NH_4_^+^ and NO_3_^−^, might be derived from large industrial sources at locations upwind. Yu et al. found that spring fugitive dusts, including soil and road dust, were more than twice as high as in other seasons in Beijing^61^.

**Figure 5:**
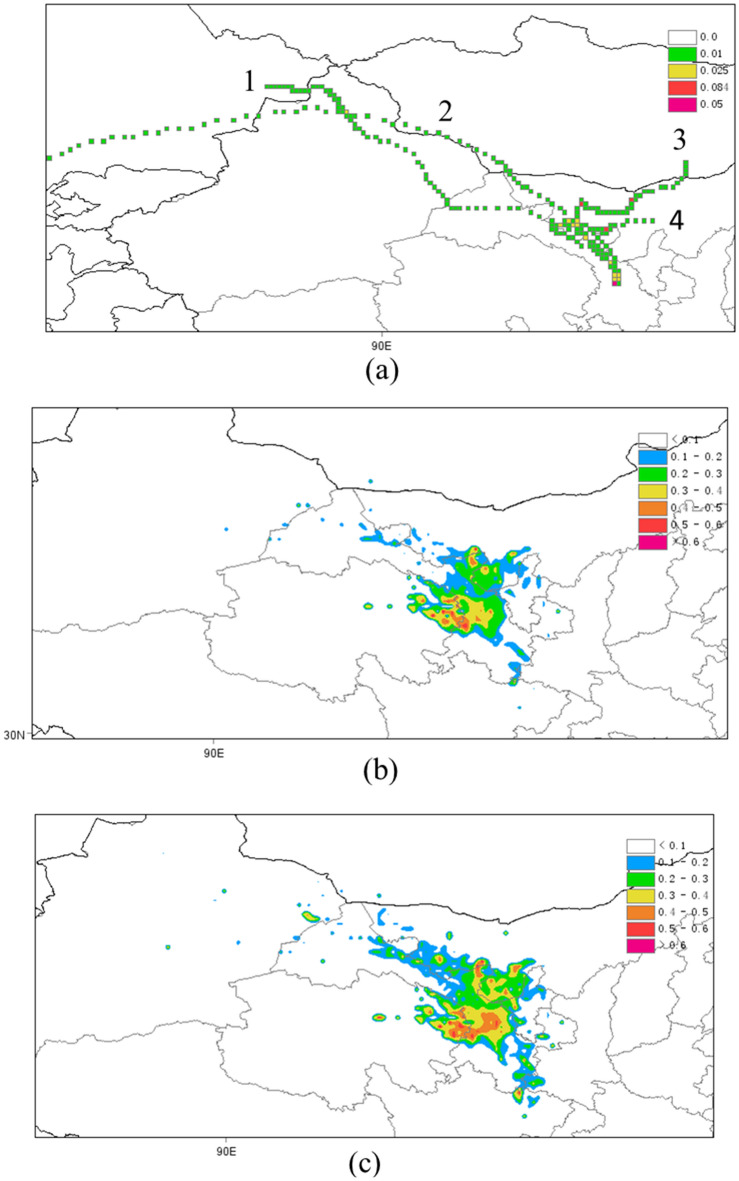
PSCF analysis for (**a**) dust storm days, (**b**) normal days, and (**c**) the entire spring in 2021 in Lanzhou, China (MeteoInfo 3.1.7 http://www.meteothink.org/downloads/index.html).

The PSCF analysis for dust storm days, normal days, and the entire spring of 2021 is presented in Fig. 5. The main trajectories that contributed most to PM_2.5_ concentrations in Lanzhou during the dust storm days are presented in Fig. 5a. The air mass of trajectory 1 originated from the desert in southeastern Kazakhstan and the Gurbantunggut Desert in northern Xinjiang and moved southeastward to Lanzhou. Trajectory 2 moved more eastward than trajectory 1, bringing dust from the deserts of western Mongolia. The air mass of trajectory 3 originated from the Gobi region in the south of Mongolia and moved southwestward through the Badain Jaran Desert in western Inner Mongolia to Lanzhou. The air mass of trajectory 4 originated from the Badain Jaran Desert in western Inner Mongolia. During the normal days, the main potential sources of PM_2.5_ were in the western and northern parts of Lanzhou (Fig. 5b). This may be due to the large amount of pollutants generated in the Xigu District, the largest petrochemical base in western China, which is located upstream of the downtown area^50^. From the spring-wide perspective, the contribution to PM_2.5_ from the northern part of Lanzhou and the Hexi Corridor was increased (Fig. 5c). Tang et al. indicated that under conditions of strong zonal jet fronts in the midlatitudes of East Asia, the Hexi Corridor is prone to low-level jets, which lead to strong sandstorms in spring^62^. Liang et al. demonstrated that the type of land (especially bare land) on the air mass movement path is a key factor that can determine the intensity of a dust storm^17^. Improving land management in windward areas of Lanzhou, especially in the Hexi Corridor, may reduce the frequency and intensity of dust storms.

The transport of pollutants would lead to the variations in concentrations of local pollutants. A study in southern Peninsular Malaysia^63^ indicated that there was a high potential for long-range transport of pollutants from heavily polluted areas, which could have a significant impact on air quality in less polluted areas. Chen et al. found that cities in the south and east of Chengdu, such as Chongqing and Neijiang, were the main sources of pollution in Chengdu^64^. Transmission of dust storms could lead to a substantial increase in the concentration of local particulate matter. A study in Seoul, Korea^41^ reported that the concentrations of PM increased during dust storm days. Xiong et al. suggested that regional sandstorms transported to Wuhan via northwest air masses in spring lead to 1.1–1.8 fold increase in the concentration of crustal elements such as Al, Ca, and Mg^65^. Turap et al. found that the concentration of PM_2.5_ in Xinjiang, China was seriously affected by dust particles from Dushanzi district and Kazakhstan in spring^66^.

**Figure 6:**
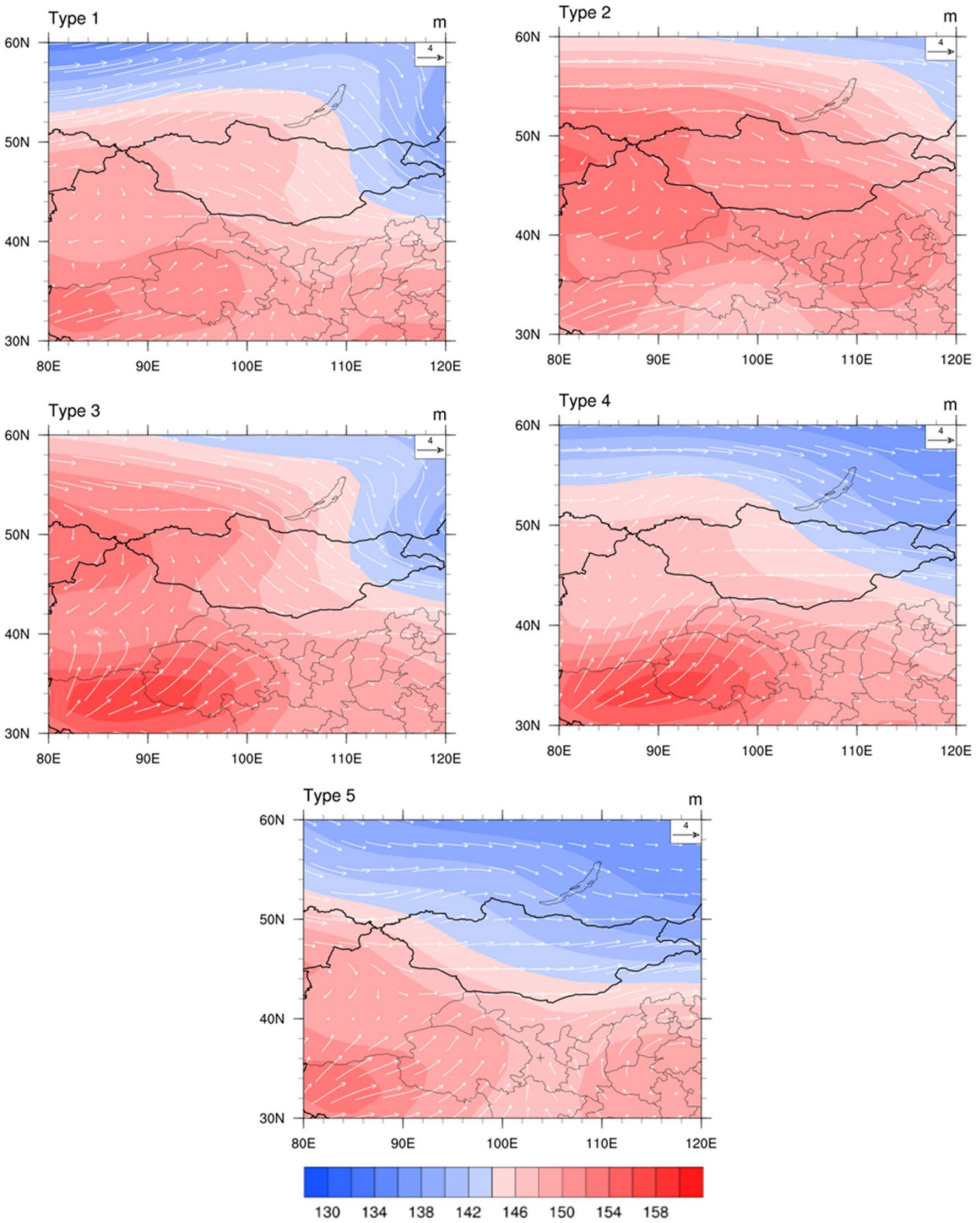
850 hPa geopotential height fields and wind vector fields of typical synoptic circulation from March 1, 2021, to May 31, 2021 (cross indicates Lanzhou, China) (NCAR Command Language https://www.ncl.ucar.edu/).

The average typical synoptic circulation from March 1, 2021, to May 31, 2021, was determined using T-PCA (Fig. 6). Type 1 was the northwest high pressure (NWH). There was a high pressure ridge in the northwestern part of Lanzhou area, and Lanzhou was affected by the Northwest (NW) wind before the ridge of high pressure. Type 2 was strong northern high pressure and southwest low pressure (NHSL). The north of Lanzhou contained a high-pressure area, and the southwest contained a low-pressure center. The atmosphere converged to the low-pressure area, and northeast winds prevailed in Lanzhou. Type 3 was northwest high pressure and southwest high pressure (NSH). The northwest and southwest of Lanzhou had two high-pressure systems. A strong north wind formed at the front of the northwest high-pressure system and a west wind formed from the southwest high-pressure system. Thus, northwest and west winds prevailed in Lanzhou in type 3. Type 4 was southwest strong high pressure (SWH^+^). Lanzhou is located in front of a high-pressure system and was, therefore, influenced by high-pressure dispersion, with prevailing west and southwest winds. Type 5 was southwest high pressure and southeast high pressure (WEH). Lanzhou is located behind a southeast high-pressure system with prevailing south winds. The frequencies of typical synoptic circulation types 1, 2, 3, 4 and 5 were 18.2, 21.7, 21.2, 19.8 and 19.0%, respectively. The typical synoptic circulation for types NWH, NHSL, and NSH were prone to dust storms. The frequency of dust storms was lower for the SWH^+^ and WEH types.

**Figure 7:**
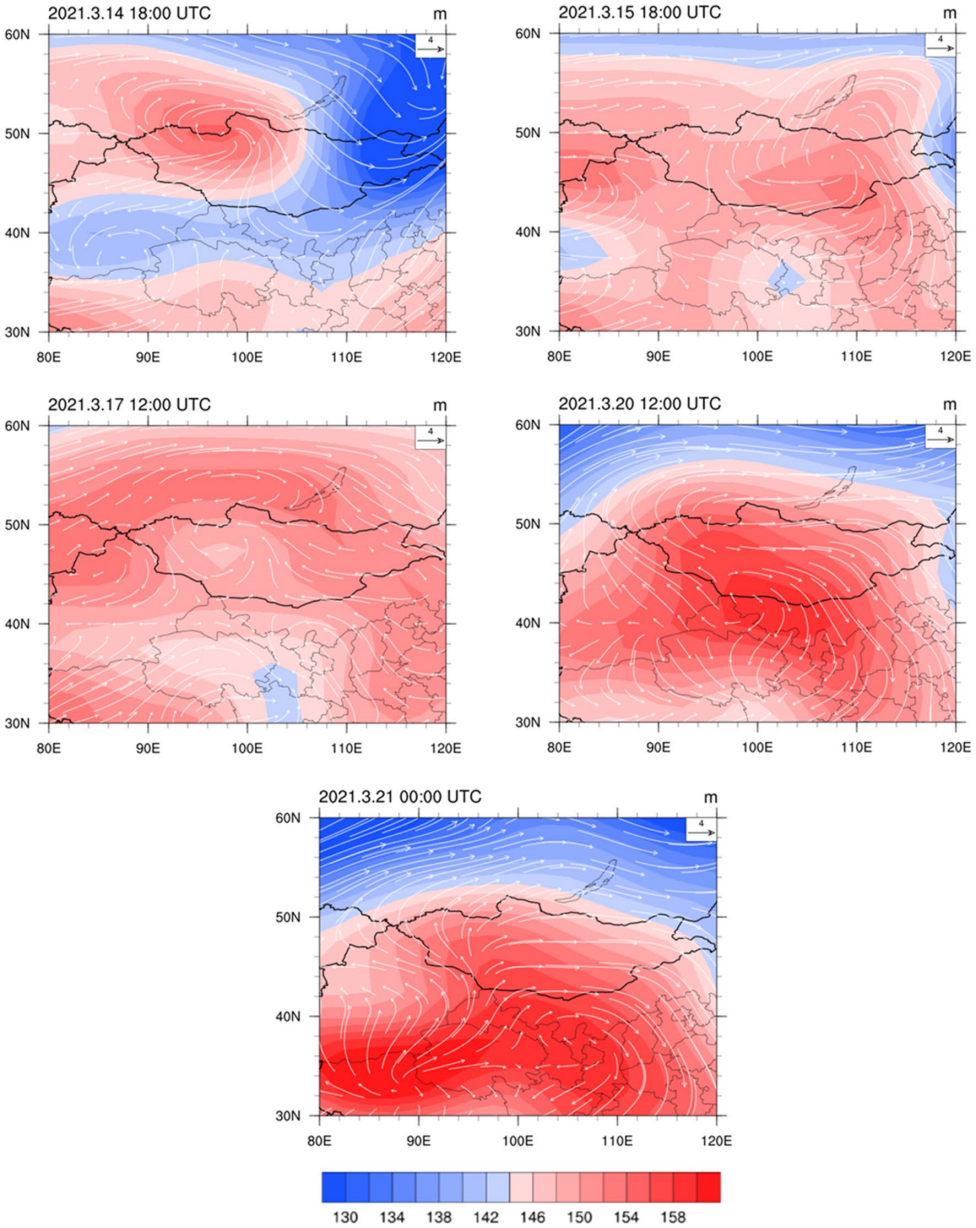
850 hPa geopotential height field and wind vector field of a typical dust storm from March 14, 2021, to March 20, 2021 (NCAR Command Language https://www.ncl.ucar.edu/).

The synoptic circulation of the most severe dust storm of the past 10 years (lasting from March 14, 2021, to March 20, 2021), which affected most parts of northern China, is presented in Fig. 7. The synoptic circulation for this dust storm process was mainly of the NSH and NHSL types. The synoptic circulation at UTC 18:00 on March 14, 2021 was of the NWH type. A cold high-pressure center occurred in the west of Mongolia, adjacent to a cyclonic system on its eastern side. A rapid accumulation of sand and dust in southern and western Mongolia and central and western Inner Mongolia was influenced by the tail end of the cyclone and the frontal part of high pressure. This sand and dust were rapidly driven into the north of China by cold air. The synoptic circulation at UTC 18:00 on March 15, 2021, and UTC 12:00 on March 17, 2021, were of the NHSL type. A low-pressure center occurred in the southwest region of Lanzhou. The convergence of the atmosphere to Lanzhou led to strong sandstorms in Lanzhou from March 15 to 17. At UTC 12:00 on March 20, 2021, a low-pressure center was still present in the southwest of Lanzhou, causing the sandstorm to continue longer in Lanzhou than in other places. The dust storm faded away when the center of the high-pressure system moved southeast.

In addition to the source of emissions, the frequency and severity of pollution events are influenced by meteorological conditions^67^. Miao et al. indicated that high-pressure systems in the southeast and east of Beijing block the flow of air to the sea, causing the polluted air of the southern industrial area to move toward Beijing^68^. Xu et al. reported that the L-shaped high synoptic circulation type is the most meteorologically adverse type, and it results in a low-pressure gradient force, weak wind speed, and rapid accumulation of PM_2.5_^31^. These weather patterns may induce the movement of atmospheric pollutants and adverse meteorological diffusion conditions, which are the key factors affecting the intensity and duration of air pollution events^13,33^^.^

Our study has some limitations. First, we measured the composition of only nine water-soluble ions in PM_2.5_ and did not include organic carbon, elemental carbon, or metal. Second, we only analyzed the chemical composition and source of PM_2.5_ in the spring dust storm season. In future research, other composition such as organic carbon, elemental carbon, and metal elements should be measured. The influence of weather conditions on the pollution in Lanzhou would be further studied on a multiyear scale. Future studies should employ sensitivity simulation to quantify the influence of different weather conditions on air quality.

## Conclusion

In this study, the emission sources and contributions of pollutants and the influence of weather situation on air pollution in the spring of 2021 in Lanzhou were comprehensively analyzed. The sources contributions of PM_2.5_ analyzed by PMF were dust (32.0%), industrial entities (29.8%), biomass combustion (13.4%), metal smelting (11.2%), secondary aerosol (10.8%), and sea salt (2.7%), respectively. During dust storm days, the concentration of Mg^2+^, Ca^2+^ and SO_4_^2−^ increased sharply. The main sources of dust were the Gobi Desert and deserts southwest and northwest of Lanzhou. The classification of weather types through T-PCA revealed that the NWH, NHSL and NSH synoptic circulation types were prone to dust storms.

## Data Availability

The datasets used in the current study are available from the corresponding author on reasonable request.
